# Genetic variability in stroke patients: *CYP2C19* polymorphisms unraveled

**DOI:** 10.1186/s12920-024-01886-8

**Published:** 2024-04-26

**Authors:** Peiyi Peng, Yingxiu Xiao, Xuehong Peng, Jianqiang Chen, Nuan Chen

**Affiliations:** 1 Shantou University Medical College, Shantou, Guangdong China; 2 Department of Neurology, The First Affiliated Hospital of Shantou University Medical College, Shantou, Guangdong China; 3 Department of Thoracic Surgery, The First Affiliated Hospital of Shantou University Medical College, Shantou, Guangdong China; 4 Department of Clinical Laboratory, The First Affiliated Hospital of Shantou University Medical College, Shantou, Guangdong China

**Keywords:** Stroke, *CYP2C19*, Gene polymorphism, Clopidogrel

## Abstract

**Objective:**

To study the distribution characteristics of *CYP2C19* polymorphisms in patients suffering from stroke in Han Chinese patients.

**Method:**

PCR and DNA microarray chip technology were used to detect the *CYP2C19* genotype of 549 patients with stroke, and the genotype, allele frequency and metabolic type of patients with different sexes, ages and types of infarctions and the independent risk factors for clopidogrel resistance were analyzed.

**Results:**

Six genotypes were detected in these 549 patients. A total of 233 (42.44%) patients had the heterozygous allele *1/*2, which was the most prevalent, followed by the homozygous wild-type allele *1/*1 (191, 34.79%). A total of 30 (5.46%) patients possessed the heterozygous allele *1/*3, and 65 (11.84%) patients had the homozygous mutant allele *2/*2. Twenty-nine (5.28%) patients had the compound heterozygous mutant allele *2/*3, and only 1 patient had the homozygous mutant allele *3/*3. The distribution of genotypes, alleles, and metabolic types did not change significantly (*P* > 0.05) by sex, age, or type of stroke. In addition, no independent risk factors for clopidogrel resistance were found in this analysis.

**Conclusion:**

The distribution of *CYP2C19* genotypes, allele frequencies, and metabolic types in patients with stroke in Han Chinese patients were not correlated with sex, age, or infarction type. The possibilities of hyperglycemia, hypercholesterolemia, hypertriglyceridemia, hypo-HDL-cholesterolemia, hyper-LDL-cholesterolemia and high blood pressure were not statistically associated with *CYP2C19* genotypes. *CYP2C19* gene polymorphism detection is recommended for patients who are available, and during treatment, the *CYP2C19* genotype can be used to guide personalized precise medication use in patients with stroke.

## Introduction

Stroke, also referred to as cerebral infarction, is a frequent clinical condition of the brain [8]. It is a substantial contributor to disability and the second most common cause of mortality worldwide [25]. Moreover, stroke-related death and disability have caused the loss of over 116 million years of healthy life each year [18]. Examples of disabilities include lack of muscle coordination, temporary or permanent bodily paralysis on one or both sides, and speech or feeding issues [25].

Stroke mainly affects the brain. Tissues inside the brain are damaged or necrotic brain tissues are formed because of a lack of blood and oxygen supply.

Brain infarction often happens when a clot (ischemic stroke) or a rupture (hemorrhagic stroke) blocks a brain artery [25]. Three different forms of stroke exist: emboli, which are produced by blood clots from other tissues,atherosclerosis of the large cerebral arteries (atherothrombotic); and occlusion of perforator arteries (lacunar) [1]. Platelet activation and aggregation are the key factors in atherosclerosis and arterial thrombosis. A study revealed that P2Y12 expressed on platelets plays an important role in the activation and aggregation of platelets [20]. Therefore, antiplatelet therapy is one of the drug therapies for stroke [12]. Aspirin and clopidogrel are the most commonly acceptable options in antiplatelet therapy,aspirin permanently inhibits cyclooxygenase (COX) enzyme activity in the prostaglandin synthesis pathway (PGH2), and clopidogrel works by inhibiting the action of adenosine diphosphate (ADP) on platelet receptors [3, 13, 21].

However, clopidogrel continues to be the most prescribed antiplatelet medication [21]. A variety of factors may contribute to this, such as its widespread acceptability, affordability, safety and minimal risk of bleeding [21]. Once clopidogrel is metabolized by cytochrome P450 enzymes, it will irreversibly inhibit P2Y12, an adenosine diphosphate (ADP) receptor on the platelet surface [6, 21, 32]. However, resistance to clopidogrel is increasingly recognized. This is because the pharmacodynamic response to clopidogrel varies widely among individuals [6]. The metabolism of clopidogrel is one of the sources of variability. It is a prodrug that is absorbed by intestinal cells and transformed into its active metabolite in hepatocytes by several CYP enzyme isoforms. This is a two-step oxidation process in which cytochrome P-450 (CYP) isoenzymes, especially *CYP2C19*, are major contributors [2, 26]. Mutations in *CYP2C19* may lower the active clopidogrel metabolite levels and reduce the effect of platelet inhibition [6]. It has been shown that *CYP2C19* is highly polymorphic and displays at least 35 alleles, including CYP2C19*1, CYP2C19*2 and CYP2C19*3. CYP2C19*1 yields normal enzyme activity, while CYP2C19*2 and CYP2C19*3 are the main mutant alleles that encode nonfunctional proteins due to single-nucleotide mutations. CYP2C19*17 (rs12248560, −806C>T) is an allele that is related to increased enzyme activity [16, 28, 37]. CYP2C19*2 (rs4244285) induces an aberrant splice site in exon 5 (c.681G>A)20, while CYP2C19*3 (rs4986893) replaces tryptophan (c.636G>A, p.W212X), resulting in a termination codon [2]. Therefore, patients carrying CYP2C19*2 and/or CYP2C19*3 are classified as intermediate metabolizers (IMs) (CYP2C19*2 or CYP2C19*3) or poor metabolizers (PMs) (CYP2C19*2 and CYP2C19*3), both of which are defined as *CYP2C19* loss-of-function allele (LoFA) carriers [24, 34]. Patients who have CYP2C19*1 are referred to as extensive metabolizers (EMs). Rapid metabolizers (RMs) are diplotypes defined by CYP2C19*1/*17, while ultrarapid metabolizers (UMs) are diplotypes defined by CYP2C19*17/*17. Although CYP2C19*17 is an increased-function allele, some data have indicated that IMs are also characterized as diplotypes with one no-function allele and one increased-function allele (for example, CYP2C19*2/*17) [4, 16, 37]. It has been reported that minor stroke patients who are LoFA carriers show a reduced response to clopidogrel, and lower levels of clopidogrel active metabolites have been found in patients carrying *CYP2C19* LoFAs [6, 33]. The U.S. Food and Drug Administration (FDA) also stated that individuals who are homozygous for no-function alleles (CYP2C19*2 and CYP2C19*3) attenuate *CYP2C19* catalytic activity and weaken the efficacy of clopidogrel [7]. Additionally, a study revealed that *CYP2C19* LoFAs are very common within Asian populations. In East Asian populations, CYP2C19*2 (frequency of 30–50%) and CYP2C19*3 (frequency of 5–10%) are the two most prevalent LoFAs. Moreover, CYP2C19*2 is the main *CYP2C19* mutation in Chinese individuals [6, 17, 33].

Currently, clopidogrel is commonly used in antiplatelet therapy in the Chaoshan area, and many patients are LoFA carriers. Unfortunately, this aspect of research is still relatively unexplored. This investigation sought to examine the outcomes of *CYP2C19* genotype identification in stroke patients in Han Chinese, analyze the genotype distribution and provide a theoretical basis for individualized precision treatment.

## Data and methods

### General data

A total of 549 Han Chinese patients with stroke admitted to the First Affiliated Hospital of Shantou University Medical College between July 2016 and August 2021 were included in this study. These individuals varied in age from 25 to 90 years old. The inclusion criteria were as follows: patients were compliant with the Chinese Guidelines for the Diagnosis and Treatment of Acute Ischemic Stroke 2018 [27], were born in three cities of Chaoshan (Shantou, Chaozhou and Jieyang), were not related to each other, and were Han Chinese. This study was approved by the Ethics Committee of the First Affiliated Hospital of Shantou University Medical College.

### Methods

#### Data collection

At admission, the patients' age, sex, infarction type, blood glucose, lipids, triglycerides, High-Density Lipoprotein, Low-Density Lipoprotein and blood pressure were noted. Approximately 2 ml of venous blood was drawn from each patient into a sterile blood collection tube with EDTA anticoagulant.

##### Reagents and instruments

The nucleic acid extraction reagent was purchased from Xiamen Zhishan Technology Co., Ltd. and was used for extraction on a LabAid820 automatic nucleic acid extraction instrument. The *CYP2C19* genetic testing kit, BR-526-24 automatic hybridizer and BE-2.0 biochip reader instrument were purchased from Shanghai BaiO Technology Co., Ltd. The BIOER PCR amplification apparatus was purchased from Hangzhou Bori Technology Co., Ltd.

##### CYP2C19 genotype testing

DNA microarray analysis and polymerase chain reaction were used to analyze *CYP2C19* gene polymorphisms. The testing method was as follows: 2 ml of blood sample was collected in an ethylenediaminetetraacetic acid (EDTA) anticoagulant tube on the day while the patient was fasting on an empty stomach. The samples were then stored in a refrigerator at 4 °C, and the DNA was extracted within 24 hours. The PCR mixture, Taq enzyme and DNA template were proportioned using the *CYP2C19* gene detection kit (DNA microarray method) provided by Shanghai Biou Technology Co., Ltd. Amplification was performed using the BIOER PCR amplification apparatus as follows: 50℃ 5min, 94℃ 5min, 35 cycles (94℃ 25sec, 48℃ 40sec, 72℃ 30sec), 72℃ 5min. The reagent was prepared according to the instructions. The chip was then removed and hybridized for color development on the BR-526-24 automatic hybridizer. Chip scanning was performed on the BE-2.0 biochip reader, and genotype image analysis was performed using *CYP2C19* genotype analysis software.

### Observation indicators

Patients suffering from stroke in the EM group, IM group, and PM group were analyzed for genotype and *CYP2C19* allele frequency.

### Statistical method

The statistical program SPSS 26.0 was used to analyze all of the data. The count data are shown as percentages (%). The chi-square (x2) test was conducted, and the results were fully analyzed. *P* <.05 was considered statistically significant.

## Results

### *CYP2C19* gene polymorphisms

**Fig. 1:**
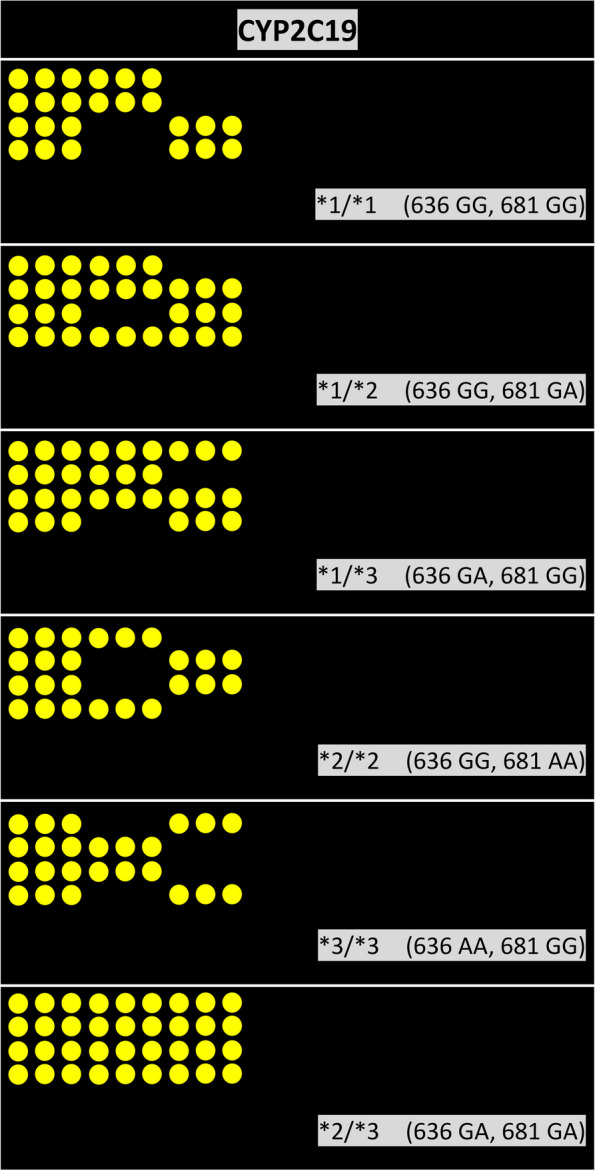
Microarray image of the genotypes of *CYP2C19*

Six categories of genotypes were found in these 549 patients, including the wild type CYP2C19*1/*1 (636GG, 681GG); heterozygous types CYP2C19*1/*2 (636GG, 681GA) and CYP2C19*1/*3 (636GA, 681GG); homozygous mutation types CYP2C19*2/*2 (636GG, 681AA) and CYP2C19*3/*3 (636AA, 681GG); and compound heterozygous mutation types CYP2C19*2/*3 (636GA, 681GA) (Fig. 1). The wild-type genotype accounted for 34.97% of patients. Of the heterozygous types, the *1/*2 genotype accounted for 42.44% of patients, and the *1/*3 genotype accounted for 5.46% of patients. Of the homozygous mutation types, the *2/*2 genotype accounted for 11.84% of patients, and the *2/*3 genotype accounted for 5.28% of patients. Only one patient was found to carry CYP2C19*3/*3. The combined frequency of the heterozygous types was the most prevalent (Table 1).

**Table 1:** *CYP2C19* gene polymorphisms.

		Number	Percentage(%)
Genotype	CYP2C19*1/*1 (636GG,681GG)	191	34.79
	CYP2C19*1/*2 (636GG,681GA)	233	42.44
	CYP2C19*1/*3 (636GA,681GG)	30	5.46
	CYP2C19*2/*2 (636GG,681AA)	65	11.84
	CYP2C19*2/*3 (636GA,681GA)	29	5.28
	CYP2C19*3/*3 (636AA,681GG)	1	0.18
Allele	CYP2C19*1	645	58.74
	CYP2C19*2	392	35.70
	CYP2C19*3	61	5.56

### *CYP2C19* gene frequency and Hardy-Weinberg equilibrium

The frequencies of *CYP2C19* polymorphic sites *∗2* and *∗3* in the patients in the current study were in accordance with the Hardy-Weinberg law of genetic equilibrium. These chosen objects are emblematic of their group (Table 2).

**Table 2:** *CYP2C19* gene frequency and Hardy-Weinberg equilibrium

Gene	Genotype	Actual number	Frequency	Theoretical number	Frequency	Allele	Number	Genotype frequency	Hardy-Weinberg equilibrium
CYP2C19*2	GG(p^2^)	222	40.44	227	41.34	G(P)	706	64.30	*X*^2^=0.8640 *P*=0.65
	GA(2pq)	262	47.72	252	45.91	A(q)	392	35.70	
	AA(q^2^)	65	11.84	70	11.84		-	-	
CYP2C19*3	GG(p^2^)	489	89.07	490	89.20	G(P)	1037	94.44	*X*^2^=0.5722 *P*=0.75
	GA(2pq)	59	10.75	57	10.50	A(q)	61	5.56	
	AA(q^2^)	1	0. 18	2	0.31		-	-	

### The frequency distribution of *CYP2C19* genotypes, alleles and clopidogrel metabolic types in different sexes

Patients in this study were classified into a male group (343 males) and a female group (206 females). The results showed that there was no statistically significant difference in the frequency distribution of *CYP2C19* genotypes between the two groups (x2=9.464, *P*=0.092). There was no statistically significant difference in the frequency distribution of *CYP2C19* alleles between the two groups (x2=2.345, *P*=0.310). However, differences in the frequency distribution of clopidogrel metabolic types were statistically significant between the two groups (x2=7.544, *P*=0.023) (Table 3).

**Table 3.** The frequency distribution of *CYP2C19* genotypes, alleles and clopidogrel metabolic types in different sexes

		Number	Male(*n*=343)	Female(*n*=206)	*X*^2^	P
Genotype	CYP2C19*1/*1	191	117 (61.26)	74 (38.74)	9.464	0.092
	CYP2C19*1/*2	233	159 (68.24)	74 (31.76)		
	CYP2C19*1/*3	30	18 (60.0)	12 (40.0)		
	CYP2C19*2/*2	65	34 (52.31)	31 (47.69)		
	CYP2C19*2/*3	29	15 (51.72)	14 (48.28)		
	CYP2C19*3/*3	1	0	1(100.0)		
Allele	CYP2C19*1	645	411(63.72)	234 (36.28)	2.345	0.310
	CYP2C19*2	392	242(61.73)	150 (38.27)		
	CYP2C19*3	61	33 (54.10)	28(45.90)		
Metabolic types	extensive metabolizers	191	117 (61.26)	74 (38.74)	7.544	0.023
	intermediate metabolizers	263	177 (67.30)	86 (32.70)		
	poor metabolizers	95	49 (51.58)	46 (48.42)		

### The frequency distribution of *CYP2C19* genotypes, alleles and clopidogrel metabolic types for different types of stroke

A total of 549 patients were divided into atherothrombotic stroke (412 patients), lacunar infarction (94 patients) and cerebral embolism (43 patients) groups. The frequency distribution of *CYP2C19* genotypes (x2=13.275, *P*=0.209), alleles (x2=4.886, *P*=0.299), and clopidogrel metabolic types (x2=5.504, *P*=0.239) did not vary statistically across the groups (Table 4).

**Table 4.** The frequency distribution of *CYP2C19* genotypes, alleles and clopidogrel metabolic types for different types of stroke

		Number	Atherothrombotic stroke(*n*=412)	Lacunar infarction(*n*=94)	Cerebral embolism(*n*=43)	X^2^	P
Genotype	CYP2C19*1/*1	191	136(71.20)	40(20.94)	15(7.85)	13.275	0.209
	CYP2C19*1/*2	233	173(74.25)	37(15.88)	23(9.87)		
	CYP2C19*1/*3	30	26(86.67)	3(10.0)	1(3.33)		
	CYP2C19*2/*2	65	53(81.54)	9(13.85)	3(4.62)		
	CYP2C19*2/*3	29	24(82.76)	4(13.79)	1(3.45)		
	CYP2C19*3/*3	1	0	1(100.0)	0		
Allele	CYP2C19*1	645	471(73.02)	120(18.60)	54(8.37)	4.886	0.299
	CYP2C19*2	392	303(77.30)	59(15.05)	30(7.65)		
	CYP2C19*3	61	50(81.97)	9(14.75)	2(3.28)		
Metabolic types	extensive metabolizers	191	136(71.20)	40(20.94)	15(7.85)	5.504	0.239
	intermediate metabolizers	263	199(75.67)	40(15.21)	24(9.13)		
	poor metabolizers	95	77(81.05)	14(14.74)	4(4.21)		

### Logistic regression analysis

The possibilities of hyperglycemia, hypercholesterolemia, hypertriglyceridemia, hypo-HDL-cholesterolemia, hyper-LDL-cholesterolemia and high blood pressure were taken as the independent variables, while the possibility of clopidogrel resistance was taken as the dependent variable. In this study, wild-type CYP2C19*1/*1 was considered clopidogrel sensitive, and the other types were considered clopidogrel resistant. These 2 variables were substituted into the logistic regression equation. None of the independent variables were shown to be independent risk factors for clopidogrel resistance by logistic regression analysis (Table 5).

**Table. 5:** Logistic regression analysis

Index	B	S.E.	Wald	df	Sig.	Exp(B)	Exp(B) 95% CI
GLU	0.172	0.189	0.824	1	0.364	1.188	0.819~1.721
CHOL	-0.177	0.382	0.215	1	0.643	0.838	0.396~1.771
TG	-0.051	0.206	0.062	1	0.804	0.950	0.634~1.424
HD	0.169	0.285	0.353	1	0.552	1.184	0.678~2.069
LD	0.159	0.437	0.133	1	0.716	1.173	0.498~2.761
High Blood Pressure	-0.063	0.230	0.074	1	0.786	0.939	0.599~1.474

## Discussion

In this study, 6 genotypes were identified among 549 patients with stroke: CYP2C19*1/*1 (636GG, 681GG) in 191 patients, accounting for 34.79%; CYP2C19*1/*2 (636GG, 681GA) in 233 patients, accounting for 42.44%; CYP2C19*1/*3 (636GA, 681GG) in 30 patients, accounting for 5.49%; CYP2C19*2/*2 (636GG, 681AA) in 65 patients, accounting for 11.84%; CYP2C19*2/*3 (636GA, 681GA) in 29 patients, accounting for 5.28%; and CYP2C19*3/*3 (636AA, 681GG) in 1 patient, accounting for 0.18%. In addition, the differences in the distribution of the different *CYP2C19* genotypes and alleles by sex were not statistically significant (*P*>0.05). This means that *CYP2C19* gene polymorphisms are not correlated with sex. However, in this study, sex was confirmed to have a significant effect on the distribution of gene-related metabolic phenotypes (*P*<0.05). Some studies have also noted the effect of sex on the clopidogrel response [10, 11], while others have denied the existence of such an effect [29]. Few studies related to the relationship between sex and clopidogrel response have been conducted,therefore, further studies are needed.

China is a country with a significant population of stroke patients, with approximately 3 million individuals experiencing their first ischaemic stroke every year [19]. However, few studies have been conducted on the association between *CYP2C19* gene polymorphisms and stroke. The associations between the *CYP2C19* genotype distribution and three different types of stroke were also investigated in this work. Although the study preliminarily revealed no statistically significant associations among these variables, the results need to be further studied due to the small number of patients who presented with cerebral embolism. According to the logistic regression analysis, extensive metabolizers were considered clopidogrel sensitive, and intermediate metabolizers and poor metabolizers were considered clopidogrel resistant.

Some studies have proposed that the genotypes CYP2C19 *1/*2, *1/*3, *2/*2 and the *CYP2C19* IM/PM phenotypes may contribute to a heightened risk of developing hypertension [5]. However, independent risk factors for clopidogrel resistance were not found in this analysis, which means that hyperglycaemia, hypercholesterolaemia, hypertriglyceridaemia, hypo-HDL-cholesterolaemia, hyper-LDL-cholesterolaemia and high blood pressure were not significantly associated with *CYP2C19* genotype.

The intermediate metabolic type was the most common clopidogrel metabolic type in the Chaoshan district, accounting for 47.9%, while the poor metabolic type, accounting for 17.3%, was less common. In the Beijing district, the intermediate metabolic type accounted for 52.48%, and the poor metabolic type accounted for 9.90%. CYP2C19*3/*3 was not detected [36]. In the Guizhou district, the most common metabolic type was the extensive metabolic type. The intermediate metabolic type accounted for 32.22%, and the poor metabolic type accounted for 10%. CYP2C19*3/*3 was also not detected [31]. Only one CYP2C19*3/*3 patient was identified in this study, which to some extent indicates that this genotype has a lower frequency of distribution in the Chaoshan area. Although the most common metabolic type differed between Shantou and Guizhou, the least common type was the same in these three districts. Notably, the genotype CYP2C19*3/*3 is rare in these three districts. One study from Indonesia showed that the majority of patients were intermediate metabolizers, and only 2.4% of them had the homozygous mutant allele (*3/*3) [22]. The most common diplotype in a Bulgarian psychiatric cohort was CYP2C19*1/*1 [14]. In Vanessa et al.’s study, CYP2C19*1/*1 was also the most common phenotype among four cohorts, a subcohort of the Admixed American superpopulation from the One Thousand Genomes Project, HGDP Native Americans, and Kaingang and Guarani living in Brazil [9].

According to reports, those with the *CYP2C19* allele with lower function are more likely to experience fatal complications and unfavourable cardiovascular events when treated with clopidogrel [16, 35]. Therefore, according to the Clinical Pharmacogenetics Implementation Consortium (CPIC), patients with *CYP2C19* IM and PM phenotypes should receive other treatments, such as ticagrelor, an ADP receptor blocker that does not require enzymatic activation. Although ticagrelor has a greater risk of bleeding and lower patient adherence, it can inhibit platelet aggregation more quickly, to a greater extent, and more consistently [15, 16, 30]. Thus, in the treatment of patients suffering from stroke, early *CYP2C19* genotype testing and expedited reporting may be beneficial to patients, which is consistent with the opinion presented in another article [23] and may help neurologists provide more individualized precision therapy to patients.

## Data Availability

The data that support the findings of this study are available on request from the corresponding author, [Nuan Chen], upon reasonable request. Due to our laboratory policy and confidentiality agreement, we cannot provide the original data. We have fully described the experimental design, analysis, and results, as well as the process of data analysis and processing. If editors and reviewers have questions about specific data, we will endeavor to provide more detailed explanations and clarifications.
